# A year in pharmacology: new drugs approved by the US Food and Drug Administration in 2021

**DOI:** 10.1007/s00210-022-02250-2

**Published:** 2022-05-11

**Authors:** Gizem Kayki-Mutlu, Zinnet Sevval Aksoyalp, Leszek Wojnowski, Martin C. Michel

**Affiliations:** 1 Department of Pharmacology, Faculty of Pharmacy, Ankara University, Ankara, Turkey; 2 Department of Pharmacology, Faculty of Pharmacy, Izmir Katip Celebi University, Izmir, Turkey; 3 Department of Pharmacology, University Medical Center, Johannes Gutenberg University, Langenbeckstr. 1, 55118 Mainz, Germany

**Keywords:** FDA, New drugs, First-in-indication, First-in-class, Next-in-class

## Abstract

The second year of the COVID-19 pandemic had no adverse effect on the number of new drug approvals by the US Food and Drug Administration (FDA). Quite the contrary, with a total of 50 new drugs, 2021 belongs to the most successful FDA years. We assign these new drugs to one of three levels of innovation: (1) first drug against a condition (“first-in-indication”), (2) first drug using a novel molecular mechanism (“first-in-class”), and (3) “next-in-class”, i.e., a drug using an already exploited molecular mechanism. We identify 21 first-in-class, 28 next-in-class, and only one first-in-indication drugs. By treatment area, the largest group is once again cancer drugs, many of which target specific genetic alterations. Every second drug approved in 2021 targets an orphan disease, half of them being cancers. Small molecules continue to dominate new drug approvals, followed by antibodies and non-antibody biopharmaceuticals. In 2021, the FDA continued to approve drugs without strong evidence of clinical effects, best exemplified by the aducanumab controversy.

## Introduction

The US Food and Drug Administration (FDA) has approved 50 new molecular entities in 2021 (U.S. Food and Administration 2022). While slightly lower than the 53 new drug approvals in 2020 (Kayki-Mutlu and Michel 2021), this remains one of the highest numbers in the past 20 years (Batta et al. 2020). In continuation of our similar analysis for 2020 (Kayki-Mutlu and Michel 2021), we here review the degree of pharmacological innovation in 2021. Discussing specific advantages and disadvantages of individual compounds against their competitors (best-in-class, best-in-indication) is beyond the scope of this article and should be left to therapeutic experts within each indication. Similarly, drug pricing, particularly in oncology, and how it relates to the clinical benefit/risk profile will not be discussed due to the complexity of the issue and the requirement for specific expertise in a therapeutic area; this a task typically reserved for Health Technology Assessment bodies in various countries. Based on these data, we discuss emerging trends in drug approvals.

## Methods

As in last year’s version (Kayki-Mutlu and Michel 2021), our analysis is based on the list of new molecular entities approved by the FDA in 2021 as communicated by the agency (U.S Food and Administration 2022). We did not include vaccines, generics or generic versions of biopharmaceuticals (“biosimilars”), or already approved drugs that received marketing authorization for an additional indication and/or in a novel formulation; newly approved drug combinations were only considered if at least one of the combination partners is a novel chemical or biological entity. Of note, other regulatory agencies may have approved the same compounds earlier than the FDA, may do so at later points in time, may choose not to approve some of these compounds, or may choose to approve compounds not approved by the FDA. Such differences may at least partly reflect that originator companies may not have filed for approval in all jurisdictions, at least not at the same time. Furthermore, the time from filing to approval may have been longer or shorter with the FDA compared to other regulatory agencies. Our focus on FDA approvals does not imply any opinion on the scientific quality of approvals by the FDA as compared to the regulatory authorities in other jurisdictions, but rather uses the FDA as a point of reference, due to its status as one of the most influential drug regulatory authorities.

We provide a short summary of mechanism of action, indication, and tolerability for each novel molecular entity and refer readers to at least one key reference on pivotal clinical evidence for further reading. The names of biopharmaceuticals (“biologics”) contain the 4-letter suffixes mandated by the FDA since 2017. They are specific to drugs deployed in the approval studies discussed in this review. Future generic versions of biopharmaceuticals will be assigned separate suffixes. This regulation accounts for the unavoidable and sometimes considerable differences in the composition, activity, and safety between the originator drug and its biosimilars, as well as among biosimilars themselves (Kliche et al. 2014). The suffixes are intended to make it easier for health care professionals to distinguish between versions of one and the same biopharmaceutical made by different manufacturers.

We assign the highest level of innovation (“first-in-indication”) to a newly approved medicine if no treatment had previously been approved for that indication. Drugs representing a novel molecular mechanism of action (“first-in-class treatments”) for conditions where other treatments had already been approved are considered the second-highest level. Drugs using the same mechanism of action as previously approved drugs in the same indication are considered as the lowest level of innovation (“next-in-class”). However, this level of innovation does not necessarily imply the absence of a clinical benefit, as new compounds within a drug class may offer advantages in efficacy, tolerability, and/or patient convenience as exemplified by fexinidazole (see section “General trends and conclusions”). Each novel drug is classified based on its innovation status as defined above (Table 1) and on the type of agent (small molecule, antibody, and peptide, and DNA/RNA-related: Table 2). As observed previously (Koster et al. 2016b, 2016a), we found a very heterogeneous reporting of data on novel entities in the peer-reviewed literature with regard to type and quantity of data being disclosed and to the number of publications. For ease of reading, we have organized our subsequent discussion by therapeutic areas.

**Table 1:** 2021 FDA drug approvals grouped by novelty as defined in “Methods.” The only first-in-indication approval, fosdenopterin for molybdenum cofactor deficiency type A, was not included in the table for ease of reading. Percentages are those of first- and next-in-class drugs with all drugs approved in 2021 taken as 100%. Where available, the International Nonproprietary Name stems in drug names have been highlighted in bold underlined based on information of the Stem Book (https://cdn.who.int/media/docs/default-source/international-nonproprietary-names-(inn)/stembook-2018.pdf?sfvrsn=32a51b3c_6&download=true) and its most recent addendum (https://cdn.who.int/media/docs/default-source/international-nonproprietary-names-(inn)/addendum-stembook2018-202108.pdf?sfvrsn=c4ec2716_7&download=true); however, corresponding information was not available for 12 out of 50 drugs

1st in class (42%)	Approved for	Next-in-class (56%)	Approved for
Aduca**numab**-avwa	Alzheimer	Asparagin**ase** erwinia chrysanthemi-rywn	Leukemia and lymphoma
Amivan**tamab**-vmjw	Non-small cell lung cancer	Asciminib	Leukemia and lymphoma
Anifro**lumab**-fnia	Systemic lupus erythematosus	Ato**gepant**	Migraine
Ava**copan**	ANCA-associated vasculitis	Aval**glucosidase** alfa-ngpt	Late-onset Pompe disease
Belumo**sudil**	Von Hippel-Lindau disease	Cabo**tegravir** and rilpi**virine**	HIV
Belzutifan	Chronic graft-versus-host disease	Casime**rsen**	Duchenne muscular dystrophy
Difeli**kef**alin	Pruritus	Dasiglucagon	Severe hypoglycemia
Efgartig**imod** alfa-fcab	Myasthenia gravis	Dostar**limab**-gxly	Endometrial cancer
Evina**cumab**-dgnb	Homozygous familial hypercholesterolemia	Drospi**renone** and **est**etrol	Contraception
Loncas**tu**xi**mab** tesirine-lpyl	Leukemia and Lymphoma	Fexi**nidazole**	African trypanosomiasis
Mariba**vir**	Cytomegalovirus infection	Fine**renone**	Chronic kidney disease associated with type 2 diabetes
Odev**ixibat**	Pruritus	Ibrexa**fung**erp	Vulvovaginal candidiasis
Pafolacianine	Diagnostic agent for ovarian cancer	Incli**siran**	Heterozygous familial hypercholesterolemia
Pegceta**cop**l**an**	Paroxysmal nocturnal hemoglobinuria	Infigra**tinib**	Cholangiocarcinoma
Sotorasib	Non-small cell lung cancer	Lonapeg**som**atropin-tcgd	Growth deficiency
Tezepe**lumab**-ekko	Asthma	Maral**ixibat**	Pruritus
Tiso**tumab** vedotin	Cervical cancer	Melphalan flufenamide	Multiple myeloma
Tralo**ki**n**umab**	Atopic dermatitis	Moboc**ertinib**	Non-small cell lung cancer
Trilacicilib	Chemotherapy-induced myelosuppression	Olanz**apine** and samid**orphan**	Schizophrenia
Veri**ciguat**	Chronic heart failure	Piflufolastat F 18	Diagnostic agent for prostate cancer
Voso**ritide**	Growth failure	Pones**imod**	Multiple sclerosis
		Ropeginterferon alfa-2b-njft	Polycythemia vera
		Serdexmethylphenidate and dexmethylphenidate	Attention deficit hyperactivity disorder
		Tepo**tinib**	Non-small cell lung cancer
		Tivoz**anib**	Renal cell carcinoma
		Umbra**lisib**	Lymphoma
		Viloxazine	Attention deficit hyperactivity disorder
		Voclosporin	Lupus nephritis

**Table 2:** 2021 FDA drug approvals grouped by drug type. The only two nucleic acid-related agents, antisense oligonucleotide casimersen and siRNA inclisiran, were not included in the table for ease of reading. Percentages are those of small molecule, antibody, and peptide drugs, with all drugs approved by the FDA in 2021 taken as 100%

Small molecule (58%)	Antibody (22%)	Peptide (16%)
Asciminib	Aducanumab-avwa	Asparaginase erwinia chrysanthemi-rywn
Atogepant	Amivantamab-vmjw	Avalglucosidase
Avacopan	Anifrolumab	Dasiglucagon
Belumosudil	Dostarlimab-gxly	Lonapegsomatropin
Belzutifan	Efgartigimod alfa-fcab	Melphalan flufenamide
Cabotegravir	Loncastuximab tesirine-lpyl	Pegcetacoplan
Difelikefalin	Sotrovimab	Ropeginterferon alfa-2b-njft
Drospirenone	Tezepelumab	Vosoritide
Evinacumab	Tisotumab vedotin	
Fexinidazole	Tixagevimab and cilgavimab	
Finerenone	Tralokinumab	
Fosdenopterin		
Ibrexafungerp		
Infigratinib		
Maralixibat		
Maribavir		
Mobocertinib		
Odevixibat		
Olanzapine and samidorphan		
Pafolacianine		
Piflufolastat		
Ponesimod		
Serdexmethylphenidate and dexmethylphenidate		
Sotorasib		
Tivozanib		
Trilaciclib		
Vericiguat		
Viloxazine		
Voclosporin		

As compared to previous years, the FDA is now often evaluating new drugs based on procedures named priority review, breakthrough therapy, fast track, and accelerated approval. Priority review designation is granted for drugs that are considered to provide a marked improvement in the therapy, diagnosis, or prevention of severe disorders, and the FDA aims to evaluate these drugs within 6 months compared to a standard review that requires 10 months (U.S. Food and Drug Administration 2018d). The breakthrough therapy designation means an accelerated evaluation process of drugs that could treat a severe condition. According to preliminary results, this novel drug exhibits more clinically relevant outcomes than current treatments (U.S. Food and Drug Administration 2018b). Fast track is an accelerated process to make critical drugs available to patients as early as possible. These patients have serious conditions such as AIDS, Alzheimer’s, heart failure, and cancer, where existing treatments are considered insufficient (U.S. Food and Drug Administration 2018c). Accelerated approval is based on a surrogate endpoint from which clinical benefit can be predicted for severe conditions that need innovative medical treatments. Post-approval clinical studies are required with drugs that receive accelerated approval (U.S. Food and Drug Administration 2018a). While these accelerated processes can bring important drugs to patients in need earlier than the standard approval procedure, they carry the risk that later evaluation based on more comprehensive datasets may lead to withdrawal, as exemplified by the withdrawal of melphalan flufenamide (Olivier and Prasad 2022) (see Oncology section).

### Oncology

As in 2020 (Kayki-Mutlu and Michel 2021), oncology dominated new drugs approvals numerically in 2021 with 15 (30%) of all approvals. Within oncology, non-small cell lung cancer had been a leading indication for new approvals in 2020 with capmatinib, lurbinectedin, pralsetinib, and selpercatinib (Kayki-Mutlu and Michel 2021). Several new drugs for treatment of non-small cell lung cancer were also approved in 2021. Among them, the antibody amivantamab and the small molecule mobocertinib target patients with the exon 20 insertion mutation of the epidermal growth factor receptor (EGFR). **Amivantamab-vmjw** is a human, bispecific monoclonal antibody and the first-in-class biopharmaceutical for the EGFR exon 20 insertion mutation-positive non-small cell lung cancer based on a priority review. Amivantamab inhibits ligand binding and disrupts the EGFR and mesenchymal-epithelial transition factor (MET) signaling pathway (Neijssen et al. 2021). Rash, allergic reactions at the infusion site, and paronychia were commonly observed using amivantamab (Brazel and Nagasaka 2021). **Mobocertinib** was approved a few months later following a priority review; while having the same target as amivantamab, it is the first orally available inhibitor of exon 20-mutated EGFR (Gonzalvez et al. 2021; Zhou et al. 2021). Mobocertinib was assessed in a phase I/II nonrandomized trial, with gastrointestinal and cutaneous complications reported as the main adverse events (AEs) (Zhou et al. 2021). **Tepotinib** is a highly selective, potent, reversible, and first oral inhibitor of the MET tyrosine kinase harboring exon 14 skipping alterations. MET is a proto-oncogene, and its abnormal signaling increases the proliferation, survival, invasion, and metastasis of tumor cells (Paik et al. 2020). Tepotinib was approved for metastatic non-small cell lung cancer in adults following a priority review, and it was generally well tolerated (Xiong et al. 2021). **Sotorasib** is the first-in-class drug targeting the G12C-mutated KIRSTEN RAT SARCOMA viral oncogene homologue (KRAS). Sotorasib was approved against non-small cell lung cancer in adults following a priority review (Blair 2021c). Grades 3 or 4 AE were observed in 11.6% of the patients with sotorasib in a phase 1 study (Hong et al. 2020b).

**Dostarlimab-gxly** is another humanized monoclonal antibody against the programmed cell death protein-1 (PD-1) receptor, which prevents its activation by the ligand PD-L1. PD-1 is a checkpoint receptor and inhibits cancer-specific immune responses. Dostarlimab was approved based on a priority review to treat mismatch repair-deficient endometrial cancer in adults. Multiple immune-related AEs were observed with dostarlimab treatment (Markham 2021a). **Tisotumab vedotin-tftv** is a first-in-class antibody–drug conjugate directed against tissue factor (Coleman et al. 2021). The tissue factor is a protein that stimulates the extrinsic coagulation cascade. It has been identified as a drug delivery target due to the high expression in multiple solid tumors. Tisotumab vedotin binds to tissue factor, which is followed by the intracellular increase of monomethyl auristatin E via proteolytic cleavage. Monomethyl auristatin E distorts the microtubules and thus causes cell cycle arrest and apoptosis (de Goeij et al. 2015). Tisotumab vedotin promotes antitumor activity with a controllable safety profile (Hong et al. 2020a), and it was approved for the treatment of cervical cancer following a priority review. The first-in-class status is based on the innovative tissue factor targeting; monomethyl auristatin E has been previously delivered as a conjugate with several other tumor-specific antibodies.

**Asparaginase erwinia chrysanthemi (recombinant)-rywn** is approved to treat acute lymphoblastic leukemia and lymphoblastic lymphoma which are hypersensitive to *Escherichia coli*-derived asparaginase. The recombinant Erwinia asparaginase addresses the supply shortage of the non-recombinant enzyme, and it is obtained via expression in *Pseudomonas fluorescens* (Lin et al. 2021). Asparaginase hydrolyzes asparagine and acts by inhibiting the growth of leukemic cells that need extracellular asparagine for their growth (Lin et al. 2021). **Asciminib** is a potent, orally bioavailable, specific STAMP (specifically targeting the ABL myristoyl pocket) inhibitor that received a priority review. It differs from previous drugs targeting this molecular target by being an allosteric ligand for a myristoyl site of BCR-ABL1 and inhibits its kinase activity (Hughes et al. 2019; Rea et al. 2021). Asciminib is approved for Philadelphia chromosome-positive chronic myeloid leukemia with a T315I mutation. In the phase III study ASCEMBL (NCT 03,106,779), the efficacy and safety of asciminib were found to be superior to bosutinib (Rea et al. 2021). **Loncastuximab tesirine-lpyl** is a first-in-class antibody–drug conjugate approved following a priority review for large B cell lymphoma treatment after combined systemic therapy in adults (Lee 2021). Loncastuximab is a humanized anti-CD19 antibody that has been identified as a delivery target in the therapy of B-cell non-Hodgkin lymphoma (Makita and Tobinai 2018). Tesirine refers to the combination of a valine-alanine cathepsin-cleavable linker and a pyrrolobenzodiazepine dimer toxin, a potent DNA crosslinker (Lee 2021). Loncastuximab tesirine has a good safety and tolerability profile (Jain et al. 2020). **Umbralisib** is an oral, highly selective phosphatidylinositol 3-kinase δ/casein kinase 1 epsilon inhibitor and inhibits cell proliferation, adhesion, and migration in lymphoma. Umbralisib was granted a priority review and approved for the treatment of marginal zone and follicular lymphoma patients who have previously received systemic therapy. Umbralisib is well tolerated (Dhillon and Keam 2021). **Melphalan flufenamide (melflufen)** is a derivative of melphalan, which was developed nearly 60 years ago and is still used for palliation in the therapy of multiple myeloma (Morabito et al. 2021). Melphalan flufenamide is an ester conjugate of melphalan with para-fluoro-l-phenylalanine, and it was approved for multiple myeloma based on a priority review but shortly afterwards withdrawn from the US market because of inferior patient survival in a phase 3 trial (Olivier and Prasad 2022). The lipophilic melphalan flufenamide easily passes across the cell membrane of neoplastic cells, where it is hydrolyzed by aminopeptidases. The released, less lipophilic melphalan becomes trapped and achieves much higher intracellular concentrations than those observed upon direct melphalan exposure (Wickstrom et al. 2017). Melphalan flufenamide is currently being tested for amyloid light-chain amyloidosis and various hematological and solid cancers (Dhillon 2021b). While cytopenias were the most common AE, alopecia and mucositis did not occur with melphalan flufenamide (Ocio et al. 2020).

**Infigratinib** inhibits the fibroblast growth factor receptor (FGFR) 1–3 reversibly and selectively (Guagnano et al. 2012). Infigratinib was approved following a priority review for previously treated, unresectable locally advanced or metastatic cholangiocarcinoma harboring an FGFR2 fusion or rearrangement (Kang 2021b). The most common AE of infigratinib treatment are hyperphosphatemia, fatigue, stomatitis, alopecia, and eye disorders (Yu et al. 2021). **Tivozanib** inhibits the vascular endothelial growth factor receptor-1, -2, -3 and c-kit potently and selectively (Kim 2017). Tivozanib prevents angiogenesis and delays carcinoma development; it was approved for the oral therapy of relapsed or refractory advanced renal cell carcinoma in adult patients following two or more systemic therapies (Kim 2017; Chang et al. 2022). The common AEs of tivozanib are hypertension, diarrhea, and skin reactions (Motzer et al. 2013).

**Belzutifan** is a small molecule and first-in-class drug that inhibits the transcription factor hypoxia-inducible factor-2α. Accumulation and activation of hypoxia-inducible factor-2α occur in von Hippel-Lindau disease, a rare genetic disorder associated with carcinoma due to inactivation of the *VHL* gene (Jonasch et al. 2021). Belzutifan was approved following a priority review for patients with von Hippel–Lindau disease who need treatment for related renal cell carcinoma, central nervous system hemangioblastomas, or pancreatic neuroendocrine tumors, but not requiring urgent surgery (Deeks 2021b). The most common AEs of belzutifan are anemia and fatigue (Deeks 2021b).

First and second-generation long-acting interferons are approved to treat chronic hepatitis B and hepatitis C (Zhu et al. 2021). Third-generation long-acting interferon **ropeginterferon alfa-2b-njft** is a mono-pegylated interferon with an increased half-life approved to treat polycythemia vera (Huang et al. 2021). The AEs of ropeginterferon alfa-2b-njft are decreased leukocytes and platelets, increased aminotransferase levels, flu-like symptoms, malaise, and arthralgia (Arya et al. 2021).

### Neurology

**Efgartigimod alfa-fcab** is a parenterally administered, humanized immunoglobulin fragment that blocks the neonatal Fc receptor. This increases the degradation of IgG antibodies. Efgartigimod is first-in-class drug approved for treating generalized myasthenia gravis in adults with increased levels of IgG antibodies against the acetylcholine receptor of the neuromuscular junction (Tice et al. 2022). AEs of efgartigimod are headache, myalgia, and reduced monocyte/lymphocyte count (Lascano and Lalive 2021).

**Ponesimod** is an orally active, selective sphingosine-1-phosphate receptor 1 modulator approved to treat relapsed multiple sclerosis (Markham 2021c). Ponesimod-induced internalization of the sphingosine-1-phosphate receptors reduces peripheral blood lymphocyte numbers (Markham 2021c). In the case of infection-driven therapy discontinuation, the immune function is restored rapidly, owing to the rapid elimination of ponesimod (Kappos et al. 2021). Before the treatment with ponesimod, a complete blood count, an electrocardiogram, liver function tests, and an ophthalmic evaluation are required (Markham 2021c). While the studies underlying the approval of ponesimod showed superior efficacy as compared to teriflunomide, it remains to be established how ponesimod compares to other sphingosine-1-phosphate receptor 1 agonists in this indication, such as the first-in-class fingolimod or ozanimod approved in 2020 (Sun et al. 2020).

Calcitonin gene–related peptide (CGRP) is secreted throughout the migraine episodes (de Vries et al. 2020). In 2020, the CGRP receptor antagonist rimegepant (Bhakta et al. 2021) had been approved for the therapy of acute migraine and prevention of episodic migraine and the anti-CGRP monoclonal antibody eptinezumab-jjmr for migraine prevention (Bhakta et al. 2021). **Atogepant** is a small-molecule CGRP receptor antagonist (Deeks 2021a) for the prophylaxis of episodic migraine in adults (Deeks 2021a). The most common AEs of atogepant were nausea, constipation, fatigue, and decreased appetite. (Deeks 2021a). The relative merit and possible differential uses of the new medications targeting the CGRP system remain to be established.

While dementia in general and Alzheimer’s disease in particular are a major burden to the afflicted patients, their relatives and caregivers, and to society at large, no new drugs had been approved for Alzheimer’s disease in the last 20 years. **Aducanumab-avwa** was approved as the first-in-class disease-modifying therapy for Alzheimer’s disease following a priority review. Aducanumab is a human monoclonal antibody administered as a monthly intravenous infusion that crosses the blood–brain barrier. There, it selectively and with high-affinity interacts with aggregated forms of amyloid-β and reduces its levels in the brain (Cummings et al. 2021). Aducanumab was effective in the early stages of Alzheimer’s disease against a surrogate endpoint. AE of aducanumab are Alzheimer-related imaging abnormality, headache, superficial siderosis, falls, and diarrhea (Abyadeh et al. 2021). Of note, the pivotal studies did not have a clinical primary endpoint, and the advisory committee of the FDA almost unanimously argued against the approval of aducanumab (Mullard 2021b).

Olanzapine, originally approved by the FDA in 1996 (Rognoni et al. 2021), is a drug for the treatment of psychotic symptoms acting primarily by mechanisms other than antagonism of dopamine D_2_ receptors, which causes weight gain, metabolic, and cardiovascular AE. A fixed-dose combination of olanzapine and the opioid receptor antagonist **samidorphan** (Paik 2021) has been approved as a once-daily oral treatment of schizophrenia and bipolar disorder. While the rationale for this combination was the hope that samidorphan would attenuate the olanzapine-associated weight gain, the efficacy and safety profile of olanzapine/samidorphan including weight gain was found to be similar to olanzapine monotherapy (Potkin et al. 2020). Therefore, its clinical value remains unclear.

**Viloxazine** is a non-stimulant medication, administered as extended-release capsules, which was approved for attention deficit hyperactivity disorder in children 6 to 17 years of age (Faraone et al. 2021); it had been available in the past in Europe as a treatment for depression. Its mechanism of action is not fully understood. It has moderate norepinephrine reuptake inhibitor, serotonin 5-HT_2B_ receptor antagonism, and 5-HT_2C_ receptor agonism properties in vitro that in combination lead to increased noradrenaline, serotonin, and dopamine levels in the prefrontal cortex in a preclinical model (Yu et al. 2020). In phase 3 studies, viloxazine extended-release exhibited clinically meaningful improvements and was well tolerated by children (Nasser et al. 2020) and adolescents with attention deficit hyperactivity disorder (Nasser et al. 2021). Dexmethylphenidate is a catecholamine reuptake inhibitor approved by the FDA for attention deficit hyperactivity disorder in 2001; it is available in immediate-release and extended-release formulation. A fixed-dose combination of the prodrug **serdexmethylphenidate** and the active compound **dexmethylphenidate** was approved in 2021 for children with attention deficit hyperactivity disorder. Serdexmethylphenidate is converted to active dexmethylphenidate in the intestine, which leads to a pharmacokinetic profile with an extended duration of action at once-daily dosing. Serdexmethylphenidate/dexmethylphenidate was well tolerated (Kollins et al. 2021). While serdexmethylphenidate/dexmethylphenidate combines the benefits of the rapid onset of the immediate-release and of the long duration of action of the marketed extended-release formulation, its clinical benefit compared to these two formulations remains to be established.

Molybdenum cofactor deficiency type A is a fatal, autosomal-recessive rare genetic disorder characterized by seizures and feeding difficulties (Kang 2021a). The deficiency results in the accumulation of toxic metabolites such as sulfites, taurine, thiosulfate, and S-sulfocysteine. **Fosdenopterin** is a synthetic derivative of endogenous cyclic pyranopterin monophosphate, which is involved in synthesizing the molybdenum cofactor. Following a priority review, fosdenopterin was approved as a first-in-indication drug to decrease the risk of mortality in molybdenum cofactor deficiency type A patients who have neurological symptoms and no other therapy options (Atwal and Scaglia 2016). The most common AEs of fosdenopterin are catheter-associated complications, vomiting, fever, cough, and infection (Kang 2021a).

Duchenne muscular dystrophy (DMD) is a genetic disorder due to dystrophin deficiency caused by mutations in the *DMD* gene (Wagner et al. 2021). **Casimersen** is an antisense oligonucleotide of the phosphorodiamidate morpholino oligomer designed to skip *DMD* gene exon 45 during mRNA processing which allows for dystrophin protein production in the skeletal muscle of DMD patients. The clinical benefit remains to be established. Casimersen was approved for the treatment of DMD following a priority review for the 8% of patients who have mutations amenable to exon 45 skipping (U.S Food and Administration 2021). Casimersen is administered by intravenous infusion and it is well tolerated (Shirley 2021).

### Lung, inflammatory, and autoimmune diseases

**Tezepelumab-ekko** is a human monoclonal antibody (IgG2λ) that inhibits thymic stromal lymphopoietin, a cytokine with an inflammatory role in airways and associated with asthma (Rochman and Leonard 2008). Tezepelumab is a first-in-class drug approved for severe asthma as an add-on maintenance treatment of adult and pediatric patients aged 12 years and older following a priority review. Tezepelumab reduces asthma exacerbations across every endpoint in a broad population of patients with severe asthma when added to standard therapy (Menzies-Gow et al. 2020; Wechsler et al. 2020). It reduces asthma exacerbations irrespective of key biomarkers and is the only drug approved for severe asthma with no phenotype (e.g., eosinophilic or allergic). It is administered via subcutaneous injection once every 4 weeks. In clinical studies, pharyngitis, arthralgia, and back pain were reported as the most common AE.

**Tralokinumab-ldrm** is a monoclonal antibody directed against interleukin-13 (IL-13). It is the first-in-class and only FDA approved drug that binds to and inhibits IL-13 specifically. It is used for the treatment of moderate-to-severe atopic dermatitis in adult patients whose disease is not adequately controlled with topical prescription therapies or when those therapies are not advisable. It is administered via weekly subcutaneous injection. Tralokinumab alone or in combination with topical corticosteroids was well tolerated (Silverberg et al. 2021; Wollenberg et al. 2021). Its common AEs include upper respiratory tract infections, conjunctivitis, injection site reactions, and eosinophilia; tralokinumab may also cause hypersensitivity reactions (Duggan 2021).

**Avacopan** is a complement 5a receptor (C5aR) antagonist. It is the first-in-class FDA-approved orally administered C5aR inhibitor and blocks C5a-mediated neutrophil activation. It is indicated for the adjunctive treatment of anti-neutrophil cytoplasmic autoantibody (ANCA)-associated vasculitis (granulomatosis with polyangiitis and microscopic polyangiitis) (Morand et al. 2020). ANCA-associated vasculitis is a multisystem autoimmune disease characterized by complement system and neutrophil overactivation leading to necrosis of blood vessels (Yates and Watts 2017). Avacopan may cause severe but rare AE such as hepatotoxicity, hypersensitivity reactions, hepatitis B virus reactivation along with its common side effects such as nausea, headache, hypertension, rash, and paresthesia (Jayne et al. 2021; Lee 2022).

Three novel drugs have been approved for the treatment of pruritus following priority reviews. **Difelikefalin** is a first-in-class selective κ opioid receptor agonist that is approved for the treatment of pruritus associated with chronic kidney disease in patients undergoing hemodialysis. It decreases itch density and improves itch-related quality of life (Fishbane et al. 2020; Sukul et al. 2021). It is administered via intravenous injection at the end of each hemodialysis treatment. Common AEs of difelikefalin treatment include diarrhea, dizziness, nausea, hyperkalemia, headache, somnolence, and mental status change. Its application has received priority review designation by the FDA. The two other anti-itch drugs are ileal bile acid transporter (IBAT) inhibitors. **Odevixibat** is a small molecule and first-in-class IBAT inhibitor approved to treat pruritus associated with progressive familial intrahepatic cholestasis that is caused by genetic mutations in patients 3 months of age and older. Odevixibat is administered orally, reduces serum bile acids, and improves pruritus assessments, body growth, and liver function markers. Liver test abnormalities, diarrhea, abdominal pain, vomiting, and fat-soluble vitamin deficiency are among its common AEs (Baumann et al. 2021; Deeks 2021c). **Maralixibat** was approved to treat cholestatic pruritus associated with Alagille syndrome in patients 1 year of age and older. Alagille syndrome is a rare disease characterized by reduced or abnormal bile ducts leading to accumulation of bile acids and ultimately to liver disease. Oral administration of maralixibat induces clinically meaningful improvements in cholestasis (Gonzales et al. 2021). It can cause serious AEs such as liver test abnormalities, fat-soluble vitamin deficiency, and some common AEs including diarrhea, abdominal pain, and vomiting (Shirley 2022).

The cyclin-dependent kinase 4 and 6 (CDK4 and CDK6) inhibitor **trilaciclib** received priority review and breakthrough designations and is a first-in-class treatment approved to prevent bone marrow suppression in patients receiving platinum/etoposide- or topotecan-containing regimens for extensive-stage small cell lung cancer. Trilaciclib infusion has beneficial effects on neutrophil- and red blood cell–related endpoints (Weiss et al. 2019; Hussein et al. 2021). The most common AEs include fatigue; low serum levels of calcium, potassium, and phosphate; increased levels of aspartate aminotransferase; headache; and pneumonia.

New treatments have also been approved for inflammatory rheumatoid diseases. The calcineurin inhibitor and immunosuppressant **voclosporin** is the first FDA-approved oral treatment for active lupus nephritis based on a priority review, and it is used in combination with mycophenolate mofetil and corticosteroids. Other calcineurin inhibitors have been approved for other indications such as prevention of organ transplant rejection, and drugs not directly targeting calcineurin but effective in the treatment of lupus nephritis including glucocorticoids and cyclophosphamide are routinely used as off-label treatments. Voclosporin treatment reduced kidney inflammation (Rovin et al. 2019, 2021). It may cause serious AEs including increased risk of cancer and infection, high blood pressure, kidney and nervous system problems, high levels of serum potassium, QT prolongation, and low red blood cell count/anemia. Some common AEs such as diarrhea, headache, anemia, cough, and urinary tract infection may also be seen.

**Anifrolumab-fnia** is a monoclonal antibody against type I interferon receptor (IFNAR) and a first-in-class drug that is approved to treat active systemic lupus erythematosus as add-on to standard therapy. It is administered through i.v. infusion once every 4 weeks. In clinical trials, anifrolumab therapy was shown to meet the primary endpoint (BILAG-based Composite Lupus Assessment response) that is associated with clinical benefit in systemic lupos erythematosus assessments and resulted in an increased number of patients with a response (Morand et al. 2020; Onuora 2020). Shingles, cough, trouble breathing, and cold symptoms such as stuffy nose, sneezing, and sore throat are among the common side effects of anifrolumab treatment.

Rho-associated coiled-coil-containing protein kinase (ROCK) was originally proposed as a potential antihypertensive drug target (Uehata et al. 1997) and later for other smooth muscle overactivity conditions such as those in the urinary bladder (Peters et al. 2006), or some cardiac pathologies (Peters and Michel 2007). ROCK also modulates inflammatory response and fibrotic processes. The ROCK inhibitor fasudil has been approved in Japan and China for some cardiovascular indications since 1995 but did not receive regulatory approval in the US or EU. **Belumosudil** is a first-in-class orally available ROCK inhibitor. It received priority review and was granted a breakthrough therapy designation for the treatment of chronic graft-versus-host disease after failure of at least two systemic therapies. Belumosudil is well-tolerated and clinically beneficial (Cutler et al. 2021; Jagasia et al. 2021). Its common AEs include infections, asthenia, nausea, diarrhea, dyspnea, cough, edema, hemorrhage, abdominal pain, musculoskeletal pain, headache, decreased phosphate, increased γ glutamyl transferase, decreased lymphocytes, and hypertension (Blair 2021a).

**Pegcetacoplan** is a first-in-class and the only FDA-approved inhibitor targeting C3, the central protein in the complement cascade. Pegcetacoplan was approved following a priority review for the treatment of paroxysmal nocturnal hemoglobinuria. It is administered subcutaneously twice a week via an infusion pump. Pegcetacoplan increases the risk of meningococcal and other serious infections caused by encapsulated bacteria. Due to this risk, it is available only under a restricted risk evaluation and mitigation strategy program (Hoy 2021). Pegcetacoplan may also lead to common AE including injection-site reactions, infections, diarrhea, breakthrough hemolysis, respiratory tract infection, viral infection, and fatigue (Hillmen et al. 2021).

### Metabolic, cardiovascular, and endocrine disorders

While several drugs are available for the treatment of hyperlipoproteinemia, they provide insufficient anti-atherosclerotic effects in some patients. Antibodies against proprotein convertase subtilisin kexin type 9 (PCSK9), alirocumab, and evolocumab were added to the therapeutic armamentarium several years ago. One of the two new anti-lipidemic drugs approved in 2021 likewise targets PCSK9. **Inclisiran** is a small interfering RNA (siRNA) directed to PCSK9 mRNA. Inhibiting PCSK9 synthesis mediates upregulation of LDL receptors on the hepatocytes, thereby lowering plasma LDL-C concentration (Rogula et al. 2021). It is a next-in-class therapy indicated for the treatment of heterozygous familial hypercholesterolemia as add-on or replacement for treatment with statins. It is administered subcutaneously every 6 months, after the initial dose and a second dose at 3 months. Inclisiran provides effective and sustained LDL-C reduction and has an acceptable safety profile (Raal et al. 2020a; Ray et al. 2020). Common AEs include injection site reaction, joint pain, urinary tract infection, diarrhea, bronchitis, pain in extremity, and dyspnea. **Evinacumab-dgnb** is an angiopoietin-like 3 (ANGPTL3) inhibitor and a first-in-class drug approved for the treatment of homozygous familial hypercholesterolemia as an orphan drug under breakthrough therapy designation and priority review. Inhibiting ANGPTL3 preserves lipoprotein lipase and endothelial lipase activities, thus reducing levels of plasma triglyceride and LDL-C (Markham 2021b). It is given by an intravenous infusion, usually once per month as an adjunct to other lipid-lowering therapies, and was shown to reduce LDL-C compared to these other treatments alone (Raal et al. 2020b). Treatment with evinacumab may cause common side reactions including flu-like symptoms, dizziness, pain in legs or arms, nausea, and fatigue.

While drug classes such as inhibitors of the renin-angiotensin system, mineralocorticoid receptor antagonists, or certain β-adrenoceptor antagonists have markedly improved survival in patients with congestive heart failure, the overall mortality remains high. Further improvement of heart failure (HF) treatment came with the neprilysin inhibitor sacubitril and the sodium-glucose transporter 2 inhibitors such as dapagliflozin (McMurray et al. 2019) and empagliflozin (Packer et al. 2020), all of which now constitute guideline-recommended treatments in heart failure with a reduced ejection fraction (McDonagh et al. 2021). Nonetheless, a medical need for further treatment options remains. The soluble guanylyl cyclase (sGC) stimulator **vericiguat** is a first-in-class drug approved for the treatment of HF following priority review. Nitric oxide-sGC-cyclic guanosine monophophate (cGMP) pathway has an important role in the cardiac function, and it is dysregulated in HF leading to impaired cardioprotection. Vericiguat binding to sGC enhances NO and cGMP activities (Markham and Duggan 2021). Oral treatment with vericiguat reduces the risk of cardiovascular death and HF hospitalization (Armstrong et al. 2020; Lang et al. 2020). Its common side effects include hypotension and anemia. Vericiguat may cause fetal harm and therefore should not be administered to pregnant women.

**Lonapegsomatropin-tcgd** is a human growth hormone used to treat growth failure caused by growth hormone deficiency in pediatric patients 1 year of age and older who weigh at least 11.5 kg. Lonapegsomatropin is a long-acting prodrug that releases somatropin identical to both the endogenous growth hormone and to the conventional, daily somatropin therapy (Thornton et al. 2021). Once-weekly subcutaneous injection of lonapegsomatropin caused greater annualized height increase compared to once-daily somatropin (Thornton et al. 2021). Viral infection, pyrexia, cough, nausea, vomiting, hemorrhage, diarrhea, abdominal pain, and arthralgia arthritis are among the AEs of lonapegsomatropin treatment. In addition, it may cause some serious side effects such as hypersensitivity reactions, increased risk of neoplasms, glucose intolerance, intracranial hypertension, fluid retention, hypoadrenalism, hypothyroidism, slipped capital femoral epiphysis, scoliosis progression, and pancreatitis.

Mineralocorticoid receptor (MR) antagonists have originally been introduced as potassium-sparing diuretics and later also for other conditions including HF (see above). In contrast to other MR antagonists that have a steroid hormone structure, **finerenone** is a non-steroidal, selective MR antagonist approved to treat patients with chronic kidney disease (CKD) associated with type 2 diabetes (T2D) based on a priority review. CKD associated with T2D are at risk for CKD progression and cardiovascular events (Thomas et al. 2015). MR overactivation is known to contribute to fibrosis and inflammation which lead to structural kidney damage (Agarwal et al. 2021). Oral finerenone treatment improved cardiovascular outcomes among the patients with T2DM and CKD (Bakris et al. 2020; Pitt et al. 2021). It reduces the risk of estimated glomerular filtration rate decline, kidney failure, non-fatal myocardial infarction, hospitalization for heart failure, and cardiovascular death. Hyperkalemia, hypotension, and hyponatremia are among the common AEs of finerenone therapy.

**Dasiglucagon** is a soluble and stable glucagon analog approved for the treatment of severe hypoglycemia in diabetic patients aged 6 years and older. It activates hepatic glucagon receptors to stimulate glycogen breakdown and glucose release resulting in an increase in blood glucose concentration (Blair 2021b). Dasiglucagon provides a rapid and sustained effect on plasma glucose (Battelino et al. 2021; Pieber et al. 2021). It is available as a single-dose autoinjector or prefilled syringe for subcutaneous injection. Common AEs include nausea, vomiting, headache, diarrhea, and injection site pain (Xu et al. 2021).

A fixed-dose combination of **drospirenone** and **estetrol** is a new oral contraceptive. While the progestin drospirenone has been introduced into medical use more than 20 years ago, estetrol is a new drug. It is a naturally occurring estrogen of unknown physiological function that is produced by human fetal liver and more selective for the estrogen receptor than other estrogens. The fixed-dose drospirenone/estetrol combination has a long half-life along with anti-androgenic and anti-mineralocorticoid properties. This novel combination demonstrates contraceptive effectiveness with a favorable bleeding profile and minimal effects on lipid profile (Creinin et al. 2021; Gemzell-Danielsson et al. 2022). It may cause irregular or painful periods, breast pain or tenderness, mood changes, and headache.

**Vosoritide** is a C type natriuretic peptide (CNP) analog and a first-in-class drug approved to increase bone growth in pediatric patients with achondroplasia under the accelerated approval and priority review, and it also was issued a rare pediatric disease priority review voucher. This genetic condition is associated with the overactivation of a gene called fibroblast growth factor receptor 3 (FGFR3) that prevents normal bone growth (Savarirayan et al. 2019). Vosoritide binds to CNP receptors, which reduces FGFR3 activity and thereby stimulates bone growth. Subcutaneous administration of vosoritide improves growth in children 5 years of age and older with open epiphyses (Savarirayan et al. 2020, 2021). The most common AEs of vosoritide include injection site reactions, arthralgia, vomiting, and hypotension.

Pompe disease is a rare disease caused by a genetic deficiency of α-glucosidase (GAA) that leads to glycogen accumulation and irreversible muscle damage resulting in respiratory dysfunction (Kohler et al. 2018). **Avalglucosidase α-ngpt** is a hydrolytic lysosomal glycogen-specific enzyme indicated for the treatment of patients 1 year of age and older with late-onset Pompe disease. It was granted fast track, priority review, breakthrough therapy, and orphan drug designations. The enzyme is conjugated with multiple mannose-6-phosphate molecules, which enhances its uptake. It was designed to increase bis-M6P levels on the molecule to enhance receptor targeting and enzyme uptake. Avalglucosidase α works by targeting the mannose-6-phosphate receptor resulting in an effective clearance of glycogen build-up in muscle cells (Dhillon 2021a). Intravenous administration of avalglucosidase α every 2 weeks reduces glycogen accumulation and improves respiratory function (Diaz-Manera et al. 2021; Kushlaf et al. 2021). Common side effects include headache, fatigue, diarrhea, nausea, arthralgia, dizziness, myalgia, pruritus, vomiting, dyspnea, erythema, paresthesia, and urticaria. Serious reactions included hypersensitivity reactions like anaphylaxis and infusion-associated reactions, including respiratory distress, chills, and raised body temperature (pyrexia). Patients susceptible to fluid volume overload or with compromised cardiac or respiratory function may be at risk for serious acute cardiorespiratory failure.

### Infectious diseases

Several SARS-CoV-2 treatments have received emergency use authorization in 2021 and are listed in a separate section below. While HIV-induced acquired immunodeficiency syndrome is no longer a deadly disease for many patients, a medical need for improved treatment remains. A fixed-dose combination of **cabotegravir** and **rilpivirine** was approved for the treatment of HIV infection under fast track and priority review. Cabotegravir is an integrase strand transfer inhibitor whereas rilpivirine is a non-nucleoside reverse transcriptase inhibitor (Markham 2020). This combination is the first injectable, complete treatment that is administered once a month to patients who are virologically suppressed and have no history of treatment failure, to replace their current therapy regimen. Before receiving the first injection, patients should receive both oral cabotegravir and rilpivirine once a day for 1 month to ensure that the drugs are well-tolerated. Clinical trials show that HIV-1 viral load is kept suppressed upon cabotegravir and rilpivirine treatment (Swindells et al. 2020; Orkin et al. 2021). The most common AEs include injection site reactions, pyrexia, fatigue, headache, musculoskeletal pain, nausea, sleep disorders, dizziness, and rash.

**Maribavir** is a cytomegalovirus (CMV) pUL97 kinase inhibitor and a first-in-class drug used for the treatment of post-transplant CMV infection that was granted orphan drug, priority review, and breakthrough therapy designations by the FDA. CMV is a β herpes virus that is latent and asymptomatic but may reactivate upon immunosuppression. In transplant patients who receive immunosuppressants, CMV can lead to serious consequences. Maribavir inhibits CMV replication via interference with several functions carried out by pUL97. Oral maribavir therapy is effective at CMV viremia clearance (Maertens et al. 2019; Avery et al. 2021). Common AEs include taste disturbance, nausea, diarrhea, vomiting, and fatigue. Maribavir was granted orphan drug and breakthrough therapy designations by the FDA.

**Fexinidazole** is a nitroimidazole with a potent *Trypanosoma brucei* activity approved for the treatment of human African trypanosomiasis (African sleeping sickness) in patients 6 years of age and older and weighing at least 20 kg following priority review. It inhibits protozoal DNA synthesis via incompletely understood mechanisms. A 10-day oral treatment with fexinidazole is the first all-oral treatment that is approved for both first stage (hemolymphatic) and second stage (meningoencephalitic) of the disease in which the parasites have crossed the blood–brain barrier, causing patients to suffer from neuropsychiatric symptoms (Kande Betu et al. 2021). Besides the advantage of oral application route, fexinidazole obviates the need for hospital treatment and is much less toxic compared to the earlier standard therapy with eflornithine and nifurtimox (Hidalgo et al. 2021). Common AEs include headache, vomiting, insomnia, nausea, asthenia, tremor, decreased appetite, dizziness, hypocalcemia, dyspepsia, back pain, upper abdominal pain, and hyperkalemia (Deeks 2019).

**Ibrexafungerp** is a next-in-class, triterpenoid antifungal agent approved under a fast track and priority review for the treatment of vulvovaginal candidiasis in adult and postmenarchal pediatric females. Clinical trials demonstrated efficacy and a favorable tolerability profile of oral ibrexafungerp therapy (Jallow and Govender 2021; Schwebke et al. 2021).

### Diagnostic agents

**Pafolacianine** is a first-in-class fluorescent imaging agent that targets the folate receptor which is overexpressed in patients with ovarian cancer. It was approved with an orphan drug, priority, and fast track designation to determine malignant lesions. It is administered intravenously prior to surgery. Pafolacianine was shown to detect additional lesions that were overlooked by standard inspection (Eskander et al. 2018; Tanyi et al. 2021). Common AEs include nausea, vomiting, abdominal pain, flushing, dyspepsia, chest discomfort, pruritus, and hypersensitivity. Patients receiving pafolacianine should avoid folate-containing supplements before administration, since folic acid may reduce the detection of cancerous tissue.

In 2020, the FDA had approved ^68^ Ga 68 PSMA-11 for the detection of prostate-specific antigen-positive lesions in men with prostate cancer (Hofman et al. 2021). In 2021, a second diagnostic agent **piflufolastat** ^**18**^**F** was approved following a priority review for positron emission tomography imaging of prostate-specific antigen-positive lesions in men with prostate cancer (Pienta et al. 2021). Piflufolastat is administered as an intravenous injection. Common AEs include headache, altered taste, and fatigue (Keam 2021).

### Emergency use authorization

Emergency use authorizations are rare and granted by the FDA only in exceptional circumstances. However, the second year of the SARS-CoV-2/COVID-19 pandemic certainly qualifies as exceptional circumstances. Emergency use authorizations are based on less comprehensive dossiers than drug approvals and are granted when the perceived harm of lack of treatment is considerably greater than the use of not fully tested medicines. While vaccines are the most effective measure to prevent SARS-CoV-2, they do not provide complete protection, and unfortunately, many refuse to get vaccinated. This establishes a need for the rapid availability of treatments of COVID-19. Following the emergency use authorization of remdesivir (Beigel et al. 2020) in 2020, several additional treatments received that authorization in 2021.

**Molnupiravir** is a small-molecule pyrimidine ribonucleoside analog that is a prodrug and converted into a synthetic cytidine nucleoside. Molnupiravir acts by introducing mutations into SARS-CoV-2 RNA (Imran et al. 2021). In the phase 3 component of the MOVe-OUT trial, oral molnupiravir was effective and well-tolerated in non-hospitalized COVID-19 patients when used within 5 days after the initiated symptoms (Jayk Bernal et al. 2021). Molnupiravir is approved for mild to moderate COVID-19.

**Nirmatrelvir** is a novel oral covalent inhibitor against SARS-CoV-2 protease (Owen et al. 2021). Nirmatrelvir was approved as a fixed-dose combination with the HIV protease inhibitor ritonavir. At the applied, low dose, ritonavir improves the pharmacokinetics of nirmatrelvir via CYP3A4 inhibition, i.e., it acts as nirmatrelvir “booster” (Drozdzal et al. 2021). In a phase 2/3 trial, nirmatrelvir/ritonavir decreased the risk of hospitalization or mortality by 89%, in mild to moderate non-hospitalized COVID-19 patients (Lange et al. 2022).

The fixed-dose combination of the monoclonal antibodies **tixagevimab** and **cilgavimab** binds and neutralizes SARS-CoV-2 and some of its variants (Dong et al. 2021). The tixagevimab/cilgavimab combination reduces the risk of a symptomatic COVID-19 in pre-exposure prophylaxis (Garcia-Lledo et al. 2021). **Sotrovimab** is an engineered humanized monoclonal antibody that binds and neutralizes SARS-CoV-2. In the COMET-IC phase II trial, sotrovimab was found to decrease the relative risk of severe or critical illness in high-risk patients with mild-to-moderate COVID-19 (Gupta et al. 2021). The monoclonal antibodies **bamlanivimab** and **etesevimab** administered as a fixed-dose combination bind and neutralize SARS-CoV-2. In a randomized phase 2/3 trial, bamlanivimab and etesevimab reduced SARS-CoV-2 burden in non-hospitalized, mild to moderate patients (Gottlieb et al. 2021). In another phase 3 trial, bamlanivimab plus etesevimab decreased the incidence of COVID-19-related hospitalization and death in high-risk ambulatory patients (Dougan et al. 2021). The bamlanivimab/etesevimab combination is indicated for mild to moderate COVID-19. Bamlanivimab and etesevimab should be administered at the earliest after a positive test result and onset of symptoms (Garcia-Lledo et al. 2021).

## General trends and conclusions

The second year of the COVID-19 pandemic demonstrates an impressive ability of the FDA to adapt the drug approval process to a long-term global emergency. The lockdowns and massive travel restrictions must have hindered the work of the FDA, most importantly the inspections of manufacturing sites. Regardless, the 50 approvals in 2021 are well in the range of the past 5 years (46–59). Nevertheless, according to press releases, a similar number of applications experienced pandemic-related delays in 2021. This suggests that under non-pandemic circumstances, the number of 2021 drug approvals could have equaled, or even surpassed, the record-breaking 59 in 2018.

These numbers suggest that the current phase of robust activity of the pharmaceutical industry continues. Commendably, by-and-large, the numbers are matched by innovation, as evidenced by almost every second drug (42%) approved in 2021 utilizing a novel molecular target (Table 1). Some of them employ a novel mechanisms of action, which mediate a drug’s pharmacodynamic effect, exemplified by the trilaciclib targets cyclin-dependent kinases 4 and 6. Some other drugs, especially antibody–drug conjugates, utilize innovative delivery targets. An illustrative example is tisotumab, which delivers the microtubule drug monomethyl auristatin E to cells expressing tissue factor.

Importantly, some of the remaining, next-in-class drugs (Table 1), demonstrate, or at least suggest, clinically meaningful advantages. A striking example is fexinidazole. Formally yet another 5-nitroimidazole-based drug, fexinidazole constitutes a milestone in the clinical management of the African trypanosomiasis. These examples demonstrate that next-in-class drugs deserve a more differentiated analysis before dismissal as “mee too” analogs.

The incentives to develop drugs for orphan diseases, which started in 1983 with the Orphan Drugs Act and intensified with the 2002 Rare Diseases Act, continue to pay off. Orphan drugs (Table 3) accounted for 52% of all FDA approvals in 2021. The significant investment of the pharmaceutical industry in orphan drugs may surprise, given the rarity of the conditions they target. However, although rare, orphan diseases are numerous and in sum affect up to 10% of the US population.

**Table 3:** 2021 FDA orphan drug approvals. Percentage is that of orphan drugs within all drugs approved by the FDA in 2021 taken as 100%

Orphan Drug (52%)	Approved indication
Asciminib	Philadelphia chromosome-positive chronic myeloid leukemia
Asparaginase erwinia chrysanthemi (recombinant)-rywn	Leukemia and lymphoma
Avacopan	Vasculitis
Avalglucosidase alfa-ngpt	Late-onset Pompe disease
Belumosudil	Chronic graft-versus-host disease
Belzutifan	Von Hippel-Lindau disease
Casimersen	Duchenne muscular dystrophy
Efgartigimod alfa-fcab	Myasthenia gravis
Evinacumab-dgnb	Homozygous familial hypercholesterolemia
Fexinidazole	Human African trypanosomiasis
Fosdenopterin	Molybdenum cofactor deficiency Type A
Infigratinib	Cholangiocarcinoma
Lonapegsomatropin-tcgd	Growth failure
Loncastuximab tesirine-lpyl	Relapsed or refractory large B-cell lymphoma
Maralixibat	Cholestatic pruritus associated with Alagille syndrome
Maribavir	CMV infection
Melphalan flufenamide	Multiple myeloma
Mobocertinib	Non-small cell lung cancer
Odevixibat	Pruritus
Pafolacianine	Diagnostic agent for ovarian cancer
Pegcetacoplan	Paroxysmal nocturnal hemoglobinuria
Ropeginterferon alfa-2b-njft	Polycythemia vera
Sotorasib	Non-small cell lung cancer
Tepotinib	Non-small cell lung cancer
Umbralisib	Marginal zone lymphoma and follicular lymphoma
Vosoritide	Achondroplasia

In 2021, the FDA continued to approve drugs without strong evidence of clinical efficacy. For example, evinacumab and inclisiran were approved based on favorable changes in surrogate lipid markers. The approval of the Alzheimer’s drug aducanumab against the advice of an external expert panel was particularly controversial. The decision may have been driven by the undoubtedly pressing and practically unmet need for Alzheimer’s drugs and the resulting public expectations. Such approvals raise medical costs, which may turn out to be unjustified, once further clinical evidence becomes available. More importantly, they may reduce the acceptance of older and clinical evidence–based drugs, if available.

Some of the 2021 new approvals confirm previously observed trends (Kayki-Mutlu and Michel 2021): Oncological treatments remain the largest group of new drugs based on indication and increasingly target tumors harboring specific mutations. Mutation-specific approvals in oncology in 2020 included avapritinib, capmatinib, selpercetinib, and tazemostat, whereas they included amivantamab, asciminib, mobocertinib, sotorasib, and tepotinib in 2021. They accounted for 4/18 and 5/15 approvals in 2020 and 2021, respectively, and allow a more targeted treatment with a potentially improved benefit/risk ratio for the targeted group as compared to the overall tumor entity. On the other hand, the associated segmentation of patient groups has implications for the design of clinical studies (smaller sample sizes) and, thereby, a thorough assessment of efficacy and tolerability as compared to other treatments. Another continuing trend is that neurology/psychiatry and infectious disease remain strong areas of innovation (Mullard 2021a). While still not being approved in large numbers, the antisense oligonucleotide casimersen (Shirley 2021) and siRNA inclisiran (Rogula et al. 2021) continue a trend for nucleic acid-related treatments as seen in the 2020 approvals of the antisense oligonucleotid viltolarsen (Iftikhar et al. 2021) and the siRNA lumasiran (Scott and Keam 2021). In a more general vein, these data testify to the ongoing innovation in medical treatment and the role of pharmacology for human wellbeing.
