# Correction to: A year in pharmacology: new drugs approved by the US Food and Drug Administration in 2021

**DOI:** 10.1007/s00210-022-02320-5

**Published:** 2022-11-06

**Authors:** Gizem Kayki-Mutlu, Zinnet Sevval Aksoyalp, Leszek Wojnowski, Martin C. Michel

**Affiliations:** 1 Department of Pharmacology, Faculty of Pharmacy, Ankara University, Ankara, Turkey; 2 Department of Pharmacology, Faculty of Pharmacy, Izmir Katip Celebi University, Izmir, Turkey; 3 Department of Pharmacology, University Medical Center, Johannes Gutenberg University, Langenbeckstr. 1, 55118 Mainz, Germany

**Correction to: Naunyn-Schmiedeberg's Archives of Pharmacology (2022) 395:867–885**

10.1007/s00210-022-02250-2

We regret to inform readers that our paper (Kayki-Mutlu et al., 2022) had mistakenly stated that tralokinumab “is administered via weekly subcutaneous injection” (p. 873). This should have read that tralokinumab “is administered as a loading dose, followed by subcutaneous injection every other week”. We apologize for any inconvenience this mistake may have caused.

